# Disparities in SARS-CoV-2 Testing in Massachusetts During the COVID-19 Pandemic

**DOI:** 10.1001/jamanetworkopen.2020.37067

**Published:** 2021-02-09

**Authors:** Scott Dryden-Peterson, Gustavo E. Velásquez, Thomas J. Stopka, Sonya Davey, Shahin Lockman, Bisola O. Ojikutu

**Affiliations:** 1 Division of Infectious Diseases, Brigham and Women’s Hospital, Boston, Massachusetts; 2 Department of Immunology and Infectious Diseases, Harvard T.H. Chan School of Public Health, Boston, Massachusetts; 3 Botswana Harvard AIDS Institute Partnership, Gaborone, Botswana; 4 Division of Global Health Equity, Brigham and Women’s Hospital, Boston, Massachusetts; 5 Department of Global Health and Social Medicine, Harvard Medical School, Boston, Massachusetts; 6 Department of Public Health and Community Medicine, Tufts University School of Medicine, Boston, Massachusetts; 7 Tufts Clinical and Translational Science Institute, Boston, Massachusetts; 8 Department of Medicine, Brigham and Women’s Hospital, Boston, Massachusetts

## Abstract

This cohort study examines disparities in severe acute respiratory syndrome coronavirus 2 (SARS-CoV-2) testing during the coronavirus disease 2019 (COVID-19) pandemic in Massachusetts.

## Introduction

Early deficiencies in testing capacity have contributed to poor control of severe acute respiratory syndrome coronavirus 2 (SARS-CoV-2),^1^ particularly among minority (ie, Black and Latino/Latina) and socioeconomically vulnerable communities.^2,3^ Allocating testing resources to locations of greatest need is important to mitigate subsequent waves of coronavirus disease 2019 (COVID-19).^4^ In the context of improved SARS-CoV-2 testing infrastructure, we examine the alignment of testing to epidemic intensity in Massachusetts.

## Methods

This cohort study was designated as not human subjects research by the Mass General Brigham institutional review board because it used anonymous, publicly available data; thus, informed consent was not sought. This study follows the Strengthening the Reporting of Observational Studies in Epidemiology (STROBE) reporting guidelines.

We compiled weekly SARS-CoV-2 molecular testing data from the Massachusetts Department of Public Health and Boston Public Health Commission for the period May 27 to October 14, 2020, following the initial COVID-19 wave. The Boston Public Health Commission reported tests of unique Boston residents, whereas the Massachusetts Department of Public Health reported total tests, including repeat testing of individuals. Consequently, we performed separate analyses for Massachusetts (351 cities and towns) and for Boston (15 neighborhoods).

We defined testing intensity as the number of SARS-CoV-2 tests performed weekly per 100 000 population and epidemic intensity as weekly test positivity. We considered optimal alignment of testing resources to be matching community ranks of testing and positivity. In communities with a testing gap (ie, the testing rank was lower than the positivity rank) in a given week, the gap was calculated as additional testing required to achieve matching ranks. For example, the testing gap for a community with the third highest positivity is the difference between its testing rate and that of the community with the third highest testing intensity.

Responses from the American Community Survey (2014-2018)^5^ were aggregated to characterize communities. Negative binomial models using robust sandwich estimators to account for repeated measures at the community level were fit to assess associations of the magnitude of the weekly testing gap with time (linearly by week), selected Centers for Disease Control and Prevention Social Vulnerability Index^6^ domains (eg, Socioeconomic Status and Minority Status/Language), and large university student population (>10% of residents). Owing to collinearity, the model of Boston neighborhoods only assessed associations with time and socioeconomic vulnerability. Two-sided Wald tests were used to assess significance at a threshold of *P* < .05. Data analysis was performed using R statistical software version 3.6.1 (R Project for Statistical Computing).

## Results

**Figure 1.**
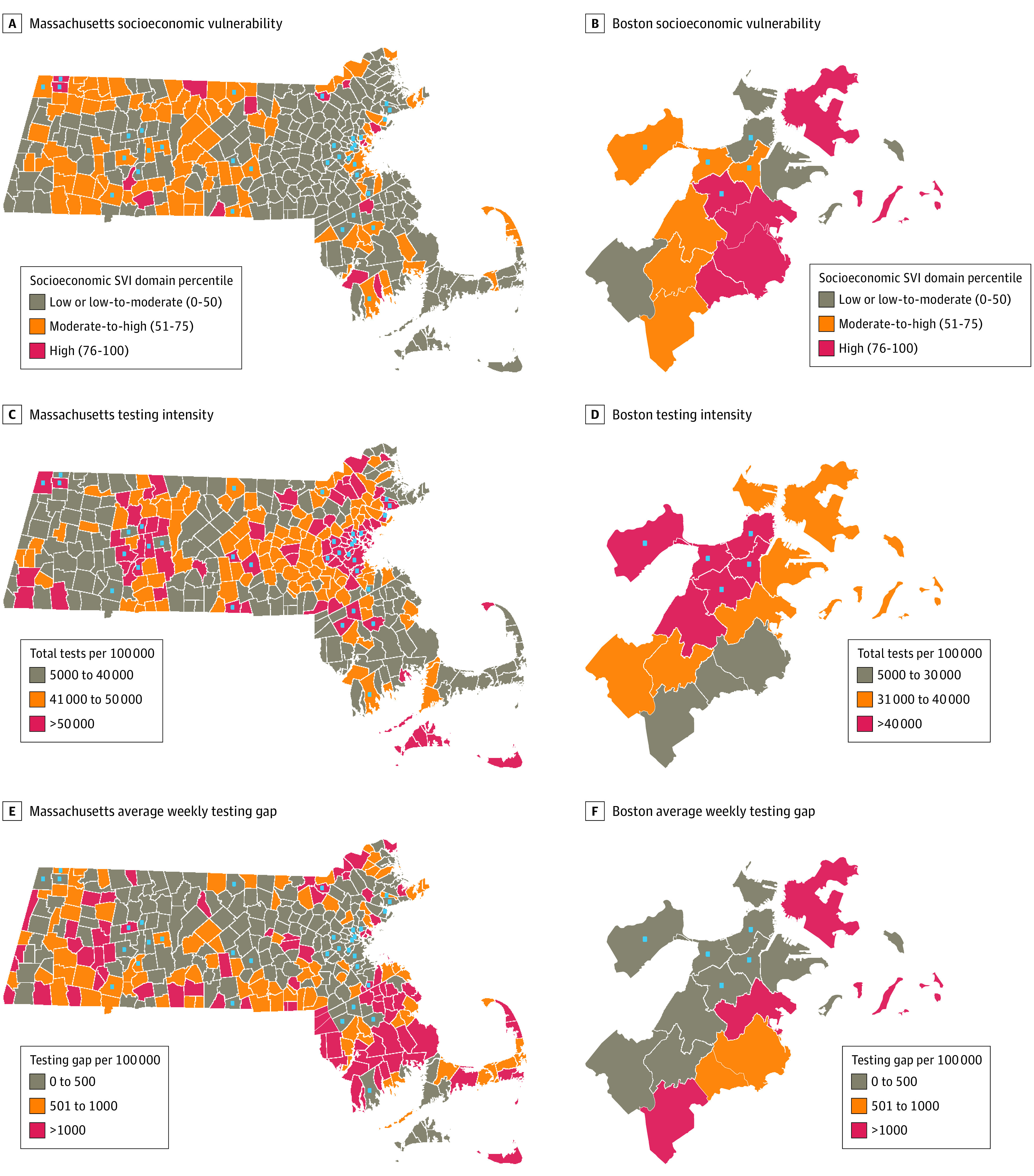
Socioeconomic Vulnerability, Severe Acute Respiratory Syndrome Coronavirus 2 (SARS-CoV-2) Testing Intensity, and SARS-CoV-2 Testing Gap Among Massachusetts Communities, May 27 to October 14, 2020 Community socioeconomic vulnerability (A and B) was estimated by the percentile from the Socioeconomic Status domain of the Centers for Disease Control and Prevention’s Social Vulnerability Index (SVI). Testing intensity (C and D) included total tests (including repeat tests in same individual) for Massachusetts but tested individuals (not including repeat testing) for Boston neighborhoods. The weekly testing gap (E and F) was calculated as the mean gap during the observation period. Blue squares indicate communities with large university student populations (>10% of residents). Data were broken into 3 categories for illustrative purposes, but statistical models considered the gap as continuous and socioeconomic vulnerability as quartiles of the US population.

During the observation period, 4 262 000 tests were reported. COVID-19 incidence (median [range], 339 [0-6670] cases per 100 000) and testing intensity (median [range], 41 000 [5350-274 000] tests per 100 000) varied considerably between communities, with observed increased testing in less socioeconomically vulnerable localities, vacation regions, and areas near universities. Considerable overlap was observed between communities with the highest socioeconomic vulnerability and those with the largest testing gaps (Figure 1).

**Figure 2.**
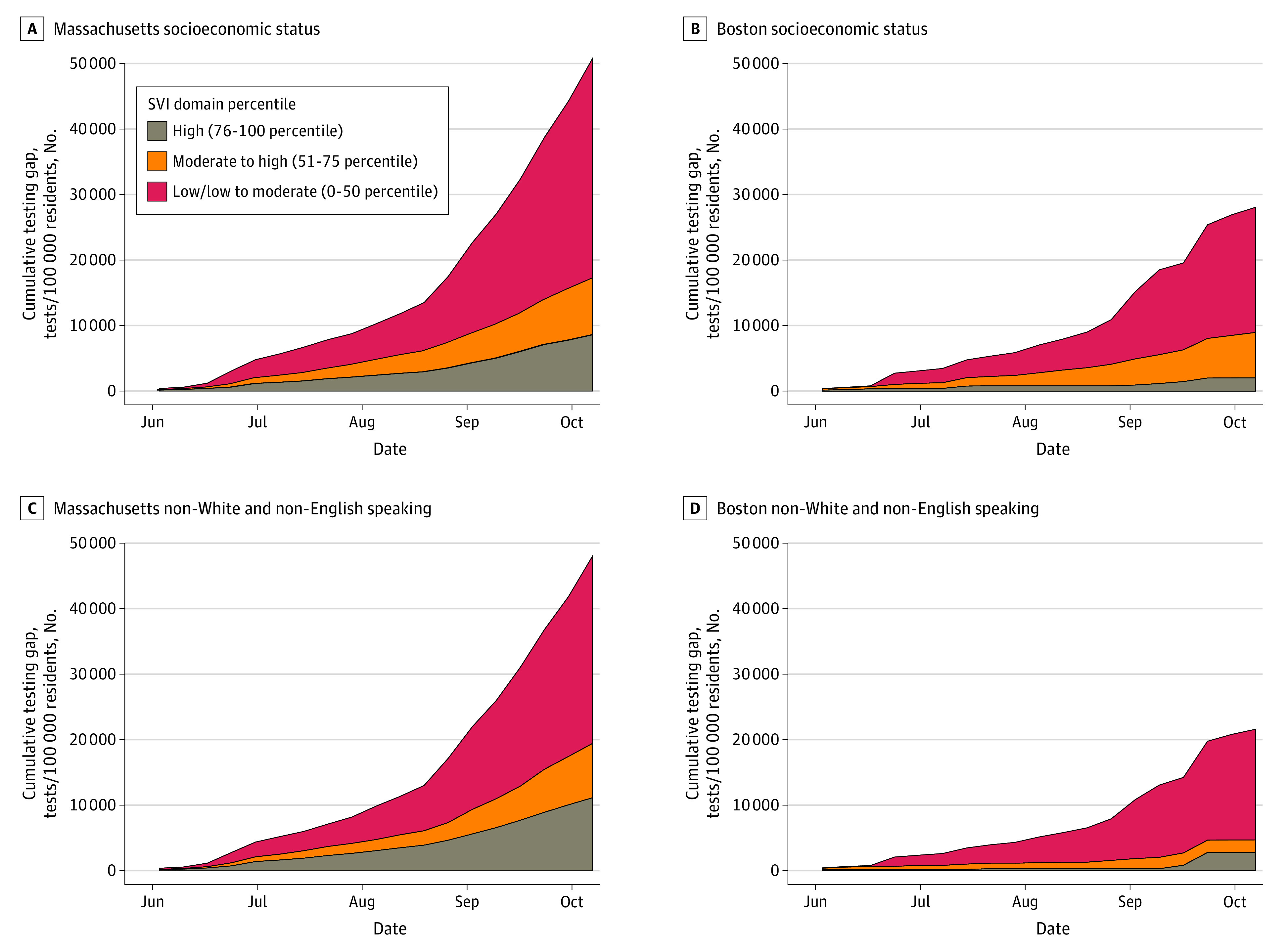
Social Vulnerability and Relative Severe Acute Respiratory Syndrome Coronavirus 2 Testing Gap Among Massachusetts Communities, May 27 to October 14, 2020 Community social vulnerability was estimated using the Socioeconomic Status and Minority Status and Language domains of the Centers for Disease Control and Prevention’s Social Vulnerability Index (SVI) aggregated to the community units used by the Massachusetts Department of Public Health (Massachusetts cities and towns) and the Boston Public Health Commission (Boston neighborhoods). Data were broken into 3 categories of percentiles of the US population.

In a multivariable model of statewide testing, the relative testing gap increased by 9.0% per week (adjusted rate ratio [aRR], 1.09; 95% CI, 1.08-1.10; *P* < .001) (Figure 2). Increasing levels of socioeconomic vulnerability were associated with increased testing gaps (aRR, 1.35 per quartile; 95% CI, 1.23-1.49; *P* < .001). Communities with the highest quartile of minority status or language vulnerability had larger testing gaps after accounting for socioeconomic status, but the difference was not significant (aRR, 1.46; 95% CI, 0.96-2.23; *P* = .08). The presence of a large university student population was associated with decreased testing gaps (aRR, 0.21; 95% CI, 0.12-0.38; *P* < .001). Similar findings were observed within Boston, with increasing testing gaps (aRR, 1.08 per week; 95% CI, 1.03-1.13; *P* = .003) and larger gaps in more socioeconomically vulnerable neighborhoods (aRR, 2.51 per quartile increase; 95% CI, 1.56-4.03; *P* < .001).

## Discussion

These analyses indicate that, despite programs to promote equity and enhance epidemic control in socioeconomically vulnerable communities, testing resources across Massachusetts have been disproportionately allocated to more affluent communities. On the basis of this analysis, we do not know how much of the testing gap is due to underutilization of testing, which may be associated with fear, anticipated stigma, and/or loss of employment. Additional limitations include the use of test positivity with varying rates of asymptomatic testing, model assumption of independence between communities, and aggregation at the community level that may underestimate disparities. Worsening structural inequities in SARS-CoV-2 testing increase the risk of another intense wave of COVID-19 in Massachusetts, particularly among socioeconomically vulnerable communities.
