# Error in Figure 2

**DOI:** 10.1001/jamanetworkopen.2021.10970

**Published:** 2021-04-23

In the Research Letter titled “Disparities in SARS-CoV-2 Testing in Massachusetts During the COVID-19 Pandemic,”^1^ published February 9, 2021, there was an error in the key in Figure 2. The colors indicating high social vulnerability and low social vulnerability were transposed in the figure key. This article has been corrected.^1^
